# Safety and initial efficacy of ablative radioembolization for the treatment of unresectable intrahepatic cholangiocarcinoma

**DOI:** 10.18632/oncotarget.28060

**Published:** 2021-09-28

**Authors:** Ricardo Paz-Fumagalli, Jacob Core, Carlos Padula, Seyed Montazeri, John McKinney, Gregory Frey, Zlatko Devcic, Andrew Lewis, Charles Ritchie, Kabir Mody, Sunil Krishnan, Beau Toskich

**Affiliations:** ^1^Department of Radiology, Division of Interventional Radiology, Mayo Clinic Florida, Jacksonville, FL 32224, USA; ^2^Division of Medical Oncology, Mayo Clinic Florida, Jacksonville, FL 32224, USA; ^3^Department of Radiation Oncology, Mayo Clinic Florida, Jacksonville, FL 32224, USA

**Keywords:** Yttrium-90, radioembolization, cholangiocarcinoma, angiography, radiation dosimetry

## Abstract

Purpose: To investigate safety, response, and survival after ablative glass microsphere ^90^Y radioembolization for unresectable intrahepatic cholangiocarcinoma.

Materials and Methods: A retrospective review of 37 radioembolizations in 28 patients treated with single compartment dose of ≥190 Gy encompassing >75% of the largest tumor was performed. Tumors were assessed for stage, morphology, and arterial supply. Response per Modified Response Evaluation Criteria in Solid Tumors (mRECIST), freedom from progression (FFP), progression-free survival (PFS), overall survival (OS), biochemical hepatic function, performance status, and adverse events were investigated.

Results: The median highest dose per patient was 256.8 Gy (195.7–807.8). Objective response at 3 months was 94.1% (complete 44.1% and partial 50%). Median OS was not reached and the 30-month OS rate was 59%, with a median follow-up of 13.4 months (5.4–39.4). FFP in the radiated field and overall FFP at 30 months were 67% and 40%, respectively. Favorable arterial supply was associated with improved OS (*p* = 0.018). Unfavorable arterial supply was associated with worse OS [HR 5.7 (95% CI 1.1–28.9, *p* = 0.034)], and PFS [HR 5.9 (95% CI 1.9–18.4, *p* = 0.002)]. Patients with mass-forming tumors had a survival benefit (*p* = 0.002). Laboratory values and performance status did not significantly change 3 months after radioembolization. Grade 3 and 4 adverse events occurred in 2 (7.1%) patients.

Conclusions: Radioembolization of unresectable intrahepatic cholangiocarcinoma with ablative intent has a high response rate, promising survival, and is well tolerated.

## INTRODUCTION

Intrahepatic cholangiocarcinoma (iCCA) is the second most common primary hepatic malignancy following hepatocellular carcinoma (HCC) [1]. Surgery is the gold standard treatment for localized iCCA, but few patients are candidates for resection at presentation and many tumors recur locally after treatment [2, 3]. Cytotoxic chemotherapy has demonstrated only a modest survival benefit, although targeted molecular and immunotherapies show potential for improved outcomes with reduced toxicity [4]. Palliative locoregional therapies are offered to select patients with unresectable iCCA and are recommended by the National Comprehensive Cancer Network guidelines [5].

Transarterial radioembolization using Yttrium-90-containing microspheres for the treatment of HCC has advanced over the past two decades from a palliative intent treatment to an ablative modality applicable as first line definitive therapy in select patients. Administering high doses of radiation to expendable volumes of liver, also known as radiation segmentectomy (two Couinaud segments or less) and lobectomy, has improved both the safety and efficacy of radioembolization. Radiopathologic analyses have supported improved pathologic necrosis rates when ablative doses are prescribed, of which 190 Gray (Gy) has shown to represent a minimal efficacy threshold [6, 7]. Whether a similar dose relationship is present with cholangiocarcinoma remains unknown. Additionally, unresectable iCCA also presents with blood supply variation and anatomic complexity, which may affect outcomes [8].

This study aimed to evaluate the initial safety and efficacy of ablative radioembolization for the treatment of unresectable iCCA, in which >75% of the tumor was treated with >190 Gy Medical Internal Radiation Dose (MIRD). An analysis of radioembolization outcomes was performed with respect to tumor blood supply characteristics.

## RESULTS

Twenty-eight patients receiving 37 radioembolizations were included for analysis. The mean age was 64.2 ± 13.1 years, with 10 males and 18 females (64%), of which 23 (82.1%) were of white race. Two patients (7.1%) had non-alcoholic steatohepatitis, 2 (7.1%) had treated hepatitis C viral infection, with the rest had no underlying liver disease. Table 1 details the tumor characteristics.

**Table 1:** Characteristics of intrahepatic cholangiocarcinomas in 28 patients

Characteristic	Patients (%)
Size (cm)	
Mean	7.3 ± SD 3.3
Median (range)	6.8 (2.0–14.0)
Distribution	
Segmental	9 (32.1)
Unilobar	8 (28.6)
Bilobar	11 (39.3)
Focality	
Solitary	16 (57.1)
Multifocal	12 (42.9)
Morphology	
Mass-Forming	22 (78.6)
Periductal-Infiltrating	5 (17.9)
Intraductal Growth	1 (3.6)
Grade of differentiation	
Well	1 (3.6)
Moderately	9 (32.1)
Poorly	5 (17.9)
Genetic Mutation	
IDH1	5 (17.9)
FGFR	3 (10.7)
Other	5 (17.9)
AJCC Stage	
Ia	3 (10.7)
Ib	4 (14.3)
II	8 (28.6)
IIIb	13 (46.4)
Quality of Vascular Conduit^*^	
Micro+/Macro+	22 (59.4)
Micro+/Macro–	8 (21.6)
Micro–/Macro–	7 (19)

^*^+: favorable, –: unfavorable.

### Radioembolization

Radioembolization dosimetry details are provided in Table 2. The median number of microsphere vials administered and individual arterial territories (angiosomes) targeted per radioembolization were 2 (range 1–4). The median segmental treatment volume was 106 cm^3^ (range 20–950) and the median lobar volume was 675 cm^3^ (range 200–1500). When a tumor required treatment of more than 1 angiosome and/or more than 1 treatment session, the highest cumulative absorbed dose was recorded. For all 28 patients, the median highest absorbed dose was 256.8 Gy (range 195.7–807.8). For segmental administrations the median highest dose was 282.9 Gy (range 210.5–807.8). For lobar administrations the median highest dose was 238.2 Gy (range 195.7–372.6). No staged bilobar or whole liver treatments were performed. Uninvolved liver was spared from radiation exposure in all patients.

**Table 2:** Dosimetry details of 37 radioembolizations in 28 patients with intrahepatic cholangiocarcinoma

Segmental radioembolization >190 Gy 18 patients, 24 treatment sessions						
Patient	Radioembolization sessions		Number of angiosomes	Volume (mL)	Activity (GBq)	Dose (Gy)
1	1		3	192	1.9	398.1
2	1		1	400	2.1	256.6
3	1		2	572	4.4	278.1
5	1		2	1015	3.6	256.9
7	1		1	31	0.2	347.4
8	2	First	4	1010	4.6	220.5
		Second	4	250	1.3	248
9	1		1	89.2	1.5	807.8
11	1		2	315	3.1	476.8
12	1		2	591	2.6	213.5
13	2	First	1	450	1.69	182.7^*^
		Second	4	1250	2.57	464.8
16	3	First	3	360	1.3	170.9^*^
		Second	2	398	1.8	219.3
		Third	3	224	1.3	289.5
18	1		4	285	1.0	221.0
19	2	First	1	220	1.1	246.1
		Second	1	85	0.8	464.1
23	1		3	299	1.5	250.2
25	1		2	300	1.3	210.5
26	1		1	123	0.6	251.9
27	2	First	3	180	1.4	362.7
		Second	1	221	1.3	247.6
28	1		2	398	2.4	287.6
**Lobar >190 Gy (*n* = 5)** **5 patients, 5 treatment sessions**						
**Patient**	**Radioembolization sessions**		**Number of angiosomes treated**	**Volume (mL)**	**Activity (GBq)**	**Dose (Gy)**
4^**^	1		1	340	2.6	372.6
6^**^	1		1	582	2.4	197.2
10^**^	1		1	1070	4.97	225.2
22	1		1	1210	9.1	363.6
24	1		1	504	2.4	220.3
**Lobar <190 Gy + segmental >190 Gy** **5 patients, 5 treatment sessions**						
**Patient**	**Radioembolization sessions**		**Number of angiosomes treated**	**Volume (mL)**	**Activity (GBq)**	**Dose (Gy)**
4^**^	1		2	675	0.9	198.6
6^**^	1		2	700	3.3	232.1
14	1		4	1636	6.6	195.7
15	1		2	978	4.9	244.2
17	1		2	650	2.6	218.0
**Lobar >190 Gy + segmental >190 Gy** **3 patients, 3 treatment sessions**						
**Patient**	**Radioembolization sessions**		**Number of angiosomes treated**	**Volume (mL)**	**Activity (GBq)**	**Dose (Gy)**
10^**^	1		2	952	4.4	225.5
20	1		2	250	1.3	254.5
21	1		3	467	2.8	290.1

^*^Those with <190 Gy MIRD in a particular treatment had more than one treatment and a cumulative dose >190 Gy to the targeted vascular territory (as stated in the Methods section). ^**^Patient 4 received 1 lobar > 190 Gy and 1 Lobar <190 Gy + segmental >190 Gy, patient 6 received 1 lobar > 190 Gy and 1 Lobar <190 Gy + segmental >190 Gy, and patient 10 received 1 lobar > 190 Gy and 1 Lobar >190 Gy + segmental >190 Gy.

### Adverse events

There were 2 (7.1%) adverse events grade 3 or greater. The grade 3 event required hospitalization two weeks after radioembolization for self-limited fever and abdominal pain. The grade 4 event was a perforated cholecystitis found on contrast-enhanced abdominal MRI 33 days after radioembolization. Direct causation from radioembolization could not established because the treated lesion was in segment VII, a vascular territory completely unrelated to the gallbladder. The bremsstrahlung single photon emission computed tomography (SPECT)/CT after radioembolization found no detectable activity in the gallbladder.

### Effect of systemic therapies

Prior, concurrent, and post radioembolization cytotoxic chemotherapy (*n* = 21; *p* = 0.438) or immunotherapy/targeted therapy (*n* = 11; *p* = 0.197) did not significantly impact overall survival (OS). Immunotherapy was given to 3, targeted therapy to 6, targeted and immunotherapy in 1, and two different targeted agents in 1.

### Response and clinical follow-up

Imaging for evaluation of response at 3 months was available for 34 of the 37 radioembolizations. Three patients had missing data because radioembolization was repeated before the 3-month scan in one, and in the other two the 3-month scan was not performed. Complete response (CR) was identified in 15 (44.1%], partial response (PR) in 17 (50%), stable disease (SD) in 1 (2.9%), and progressive disease (PD) in 1 (2.9%) patients. This yielded an overall response rate (CR and PR) of 94.1% and a disease control rate (CR, PR or SD) of 97.1%. Pre and 3-month post-treatment Albumin-Bilirubin (ALBI) scores did not differ significantly (*p* = 0.22). Baseline ALBI grade 1 was observed in 31/37 (83.8%) and ALBI grade 2 in 6/37 radioembolizations (16.2%). ALBI grade changes increased from grade 1 to 2 in 7/37 (19%), grade 2 to 3 in 1/37 (3%), and decreased from grade 2 to 1 in 1/37 (3%) radioembolizations. Changes in Model for End-Stage Liver Disease score (MELD) (*p* = 0.16), Child-Pugh (CP) class (*p* = 0.25), and Eastern Cooperative Oncology Group (ECOG) performance status (*p* = 0.22) scores were not significant, but elevated baseline CA 19-9 decreased significantly (*p* = 0.041). Six patients (21.4%) were down-staged to resection after radioembolization. Surgical approaches included one right hepatectomy, one right hepatectomy and non-anatomic segment II resection, two extended right hepatectomies, one central hepatectomy, and one had left hepatectomy with partial resection of segment I.

### Freedom from progression

**Figure 1:**
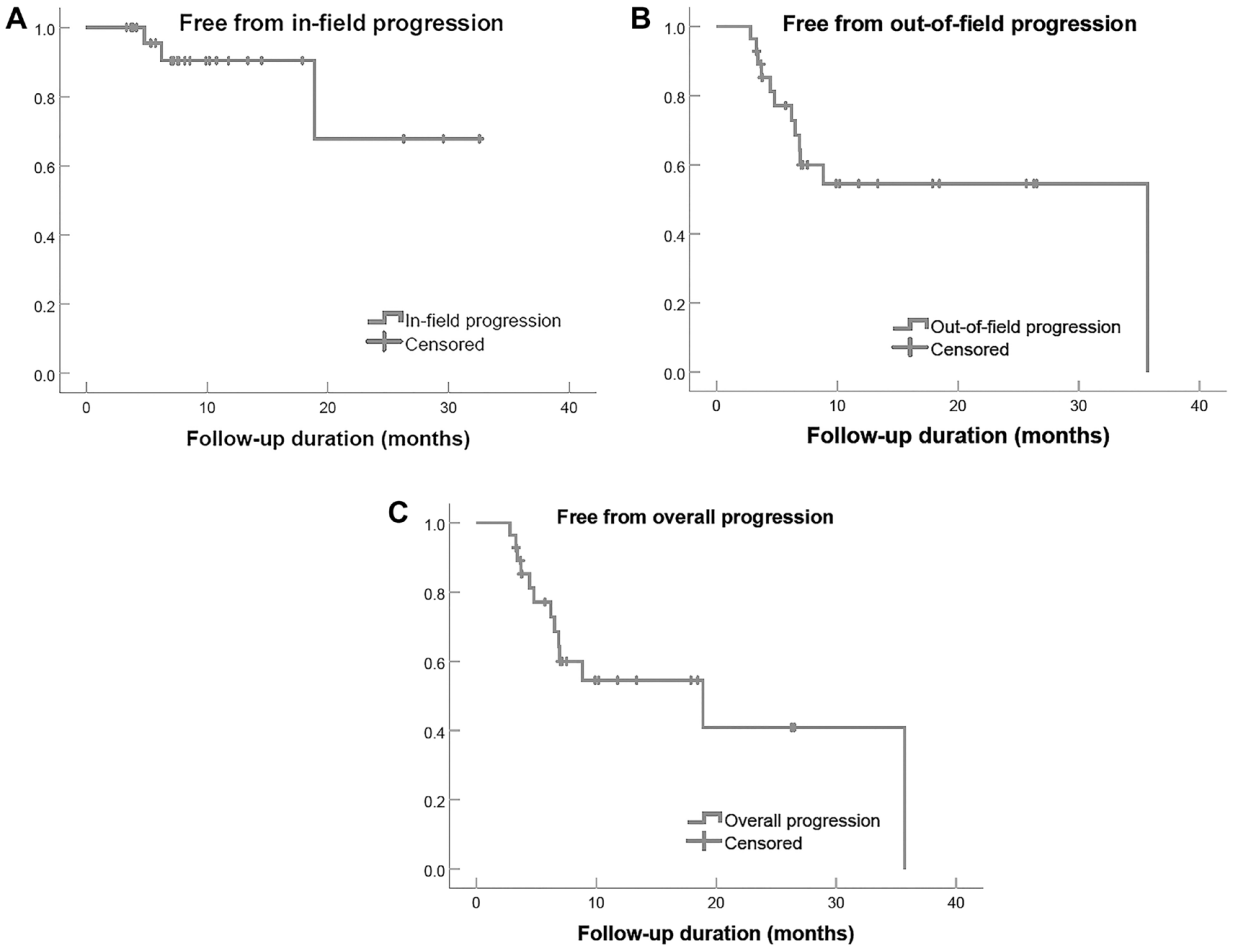
Freedom from progression in 28 patients with intrahepatic cholangiocarcinoma treated with radioembolization, categorized by in-field (**A**), out-of-field (**B**), and overall progression (**C**).

Table 3 and Figure 1 detail the overall, in-field, and out-of-field freedom from progression (FFP) at 30-months after therapy. In-field progression occurred in 3 patients (10.7%) while out-of-field progression occurred in 12 (42.9%). Solitary and segmental disease correlated significantly with better FFP (*p* = 0.006 and 0.015, respectively).

**Table 3:** Freedom from progression after radioembolization for intrahepatic cholangiocarcinoma in 28 patients

	Freedom from Progression %				
	6 months	12 months	18 months	24 months	30 months
Location of tumor progression					
In-field	96	89	89	67	67
Out-of-field	77	51	51	51	51
Overall	77	51	51	40	40

### Survival analysis

**Figure 2:**
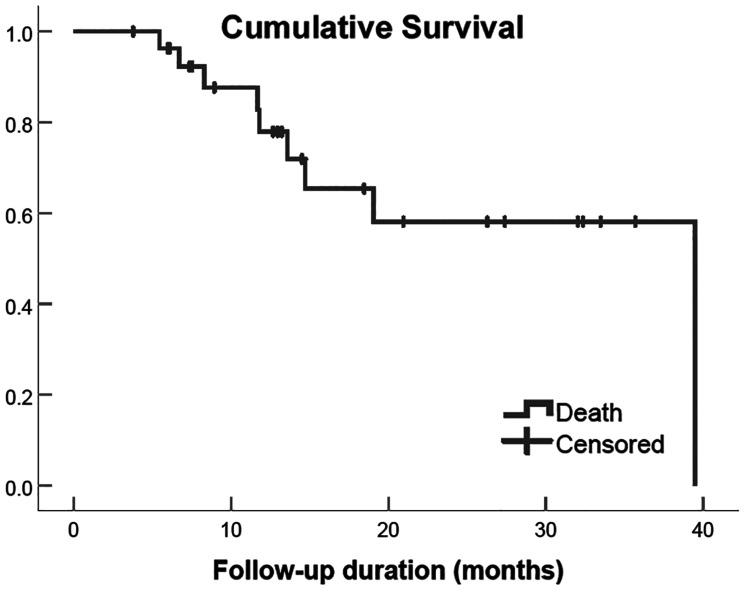
Overall survival of 28 patients with iCCA treated with ablative radioembolization.

During the median follow-up time of 13.4 months (range 5.4–39.4), 9 of 28 patients died (32.1%). OS at 3 years was 59% (Table 4 and Figure 2). The OS was not statistically different between segmental, unilobar, and bilobar tumor distribution, but bilobar tumor had a trend for greater risk of death with a hazard ratio (HR) of 2.5 (95% CI 0.6, 10.4 and *p* = 0.218). Mass-forming tumor morphology was associated with significant survival benefit (Table 5).

**Table 4:** 3-year overall and progression-free survival in 28 patients after radioembolization for intrahepatic cholangiocarcinoma

Parameter	Overall survival (%) in Months						Progression-Free survival (%) in Months					
	6	12	18	24	30	*p*-value	6	12	18	24	30	*p*-value
**All Patients**	96	78	67	59	59		74	45	40	25	25	
**Anatomic Distribution**						0.403						**0.003**
Segmental (*n* = 9)	88	88	88	59	59		88	88	88	59	59	
Unilobar (*n* = 8)	100	83	83	83	83		878	39	39	39	39	
Bilobar (*n* = 11)	100	68	43	43	43		55	18	9	0	0	
**Tumor Focality**						0.112						**0.002**
Solitary (*n* = 16)	93	85	85	85	85		87	63	63	63	63	
Multifocal (*n* = 12)	100	71	49	37	37		58	25	17	0	0	
**Tumor Morphology**						**0.002**						0.382
Mass Forming (*n* = 22)	95	90	77	68	68		67	52	46	29	29	
Periductal/intraductal (*n* = 6)	100	0	0	0	0		100	11	0	0	0	

**Table 5:** Univariate analysis of tumor characteristics of 28 patients with intrahepatic cholangiocarcinoma treated with radioembolization

Parameter	Overall survival		Progression-Free survival	
	HR (95% CI)	*p* value	HR (95% CI)	*p* value
Size				
(1 cm increments)	0.93 (0.73, 1.18)	0.553	**1.19 (1.01, 1.41)**	**0.040**
<5 cm versus ≥5 cm	0.28 (0.06, 1.30)	0.105	1.12 (0.41, 3.10)	0.824
Distribution				
Segmental or unilobar	Ref		Ref	
Bilobar	2.46 (0.59, 10.36)	.218	**5.02 (1.73, 14.57)**	**0.003**
Focality				
Solitary	Reference		Reference	
Multifocal	3.40 (0.68, 16.86)	0.135	**4.44 (1.55, 12.73)**	**0.005**
Morphology				
Mass Forming	Ref		Ref	
Periductal Infiltrating and Intraductal growth	**11.39 (1.72, 75.63)**	**0.012**	1.69 (0.51, 5.60)	0.387
Metastatic Adenopathy	2.21 (0.49, 9.89)	0.301	2.90 (0.99, 8.54)	0.053
AJCC Stage				
Ia or Ib	Ref		Ref	
II	2.11 (0.19, 23.42)	0.542	7.81 (0.89, 68.59)	0.064
IIIb	2.77 (0.32, 23.83)	0.353	14.18 (1.66, 121.40)	0.016
Vascular Conduit				
Macro+ and Micro+	Ref		Ref	
Macro– and/or Micro–	**5.7 (1.1–28.9)**	**0.034**	**5.9 (1.9–18.4)**	**0.002**

Abbreviations: Macro+: favorable macrovascular; Micro+: favorable microvascular; Macro–: unfavorable microvascular; Micro–: unfavorable microvascular.

The median progression free survival (PFS) for the entire cohort was 8.8 months (95% CI, 1.0–16.7). Solitary tumor had a significantly greater PFS compared to multifocal disease (*p* = 0.002). Segmental disease was a significant predictor of greater PFS (*p* = 0.003). Univariate analysis of tumor characteristics demonstrated significant influence on OS and PFS (Table 5). Combined periductal infiltrating and intraductal types had a significantly increased hazard for worse OS compared to mass-forming tumor (HR, CI, *p* value). Survival for stage IIIb compared to earlier stages combined was not significant (*p* = 0.42). The PFS was significantly worse for every 1 cm tumor diameter increase (*p* = 0.040), bilobar versus segmental or unilobar distribution (*p* = 0.003), and multifocal versus solitary tumor (*p* = 0.005).

### Tumor arterial supply (vascular conduit) qualitative analysis

**Figure 3:**
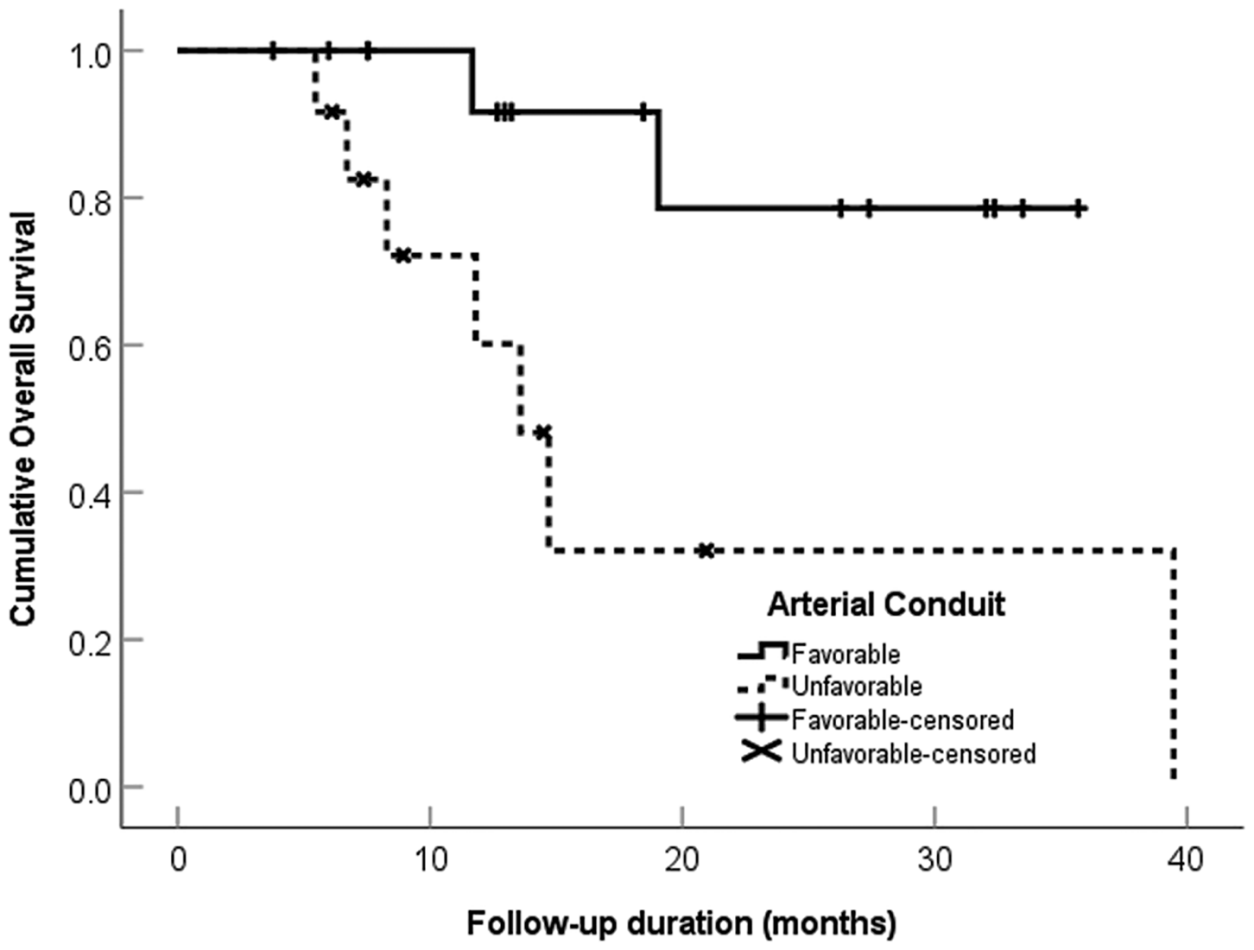
Overall survival categorized by arterial conduit favorability in 28 patients with intrahepatic cholangiocarcinoma treated with ablative radioembolization.

**Figure 4:**
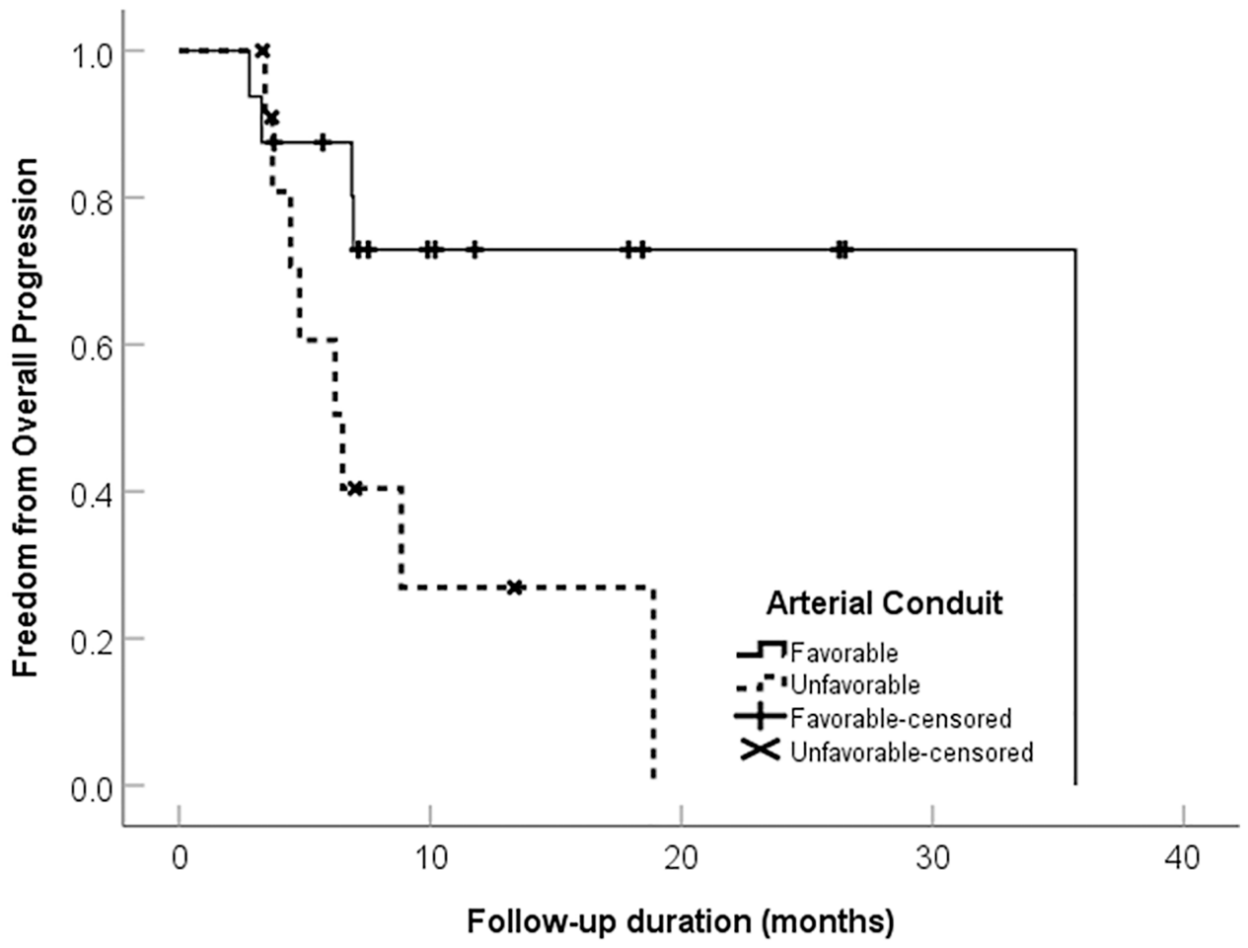
Freedom from progression categorized by arterial conduit favorability in 28 patients with intrahepatic cholangiocarcinoma treated with ablative radioembolization.

**Figure 5:**
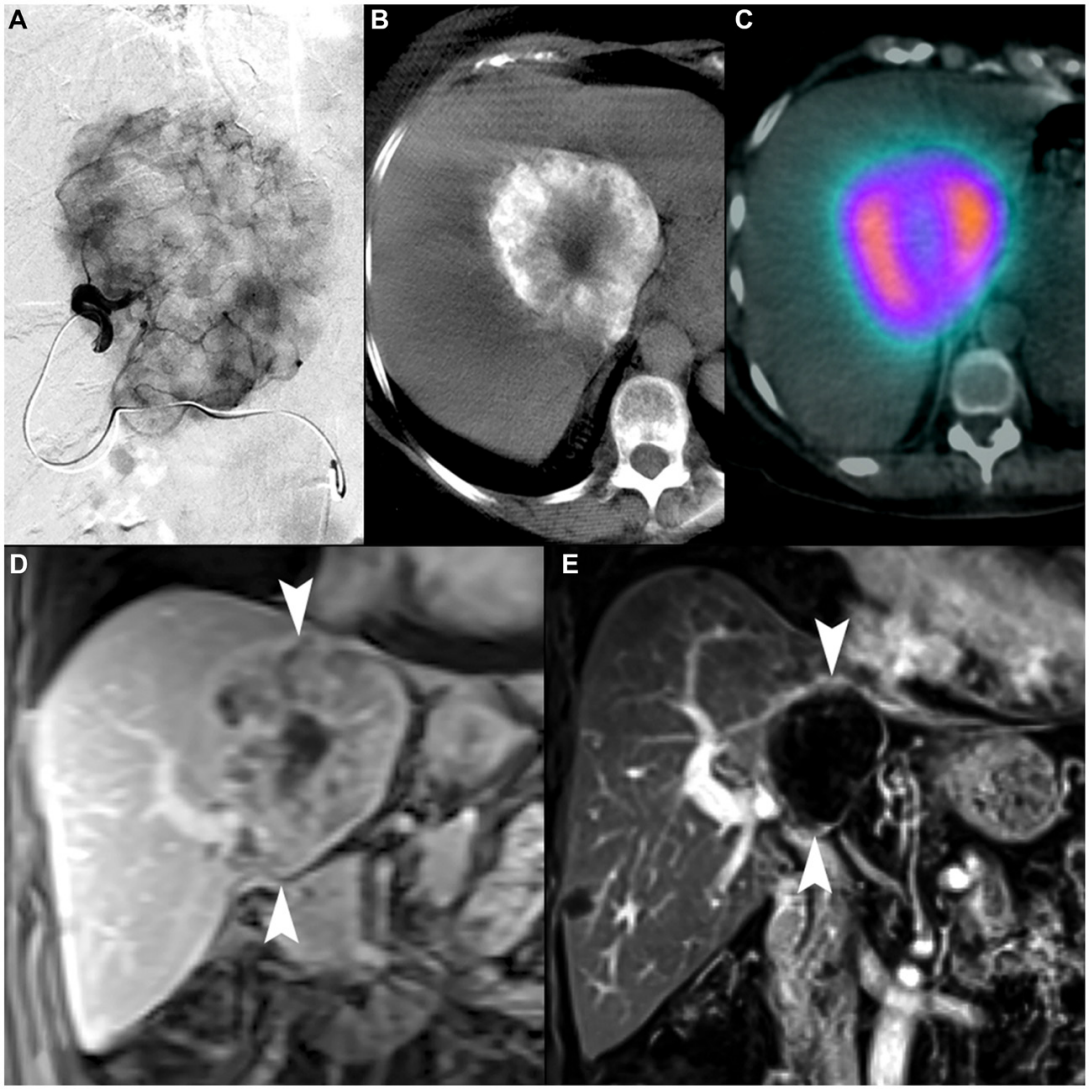
70-year-old female with intrahepatic cholangiocarcinoma. (**A**) Selective arteriography of a 9.2 cm iCCA demonstrating favorable macrovascular conduit for radioembolization. (**B**) Cone-beam computed tomography showed complete tumor coverage within two angiosomes (second not shown). (**C**) ^99m^Tc-MAA SPECT/CT demonstrated highly conformal tracer distribution with minimal exposure to non-tumoral liver. The MAA uptake within tumor (including the lower-activity necrotic center) demonstrates an example of favorable microvascular conduit due to overall relative uptake to normal liver parenchyma. (**D**) Coronal contrast-enhanced MRI image demonstrates the tumor at initial presentation (arrowheads). (**E**) Coronal contrast-enhanced MRI 3 years after single-session radioembolization with a dose of 476.8 Gy shows a persistent mRECIST complete response and contraction to 4.2 cm (arrowheads).

**Figure 6:**
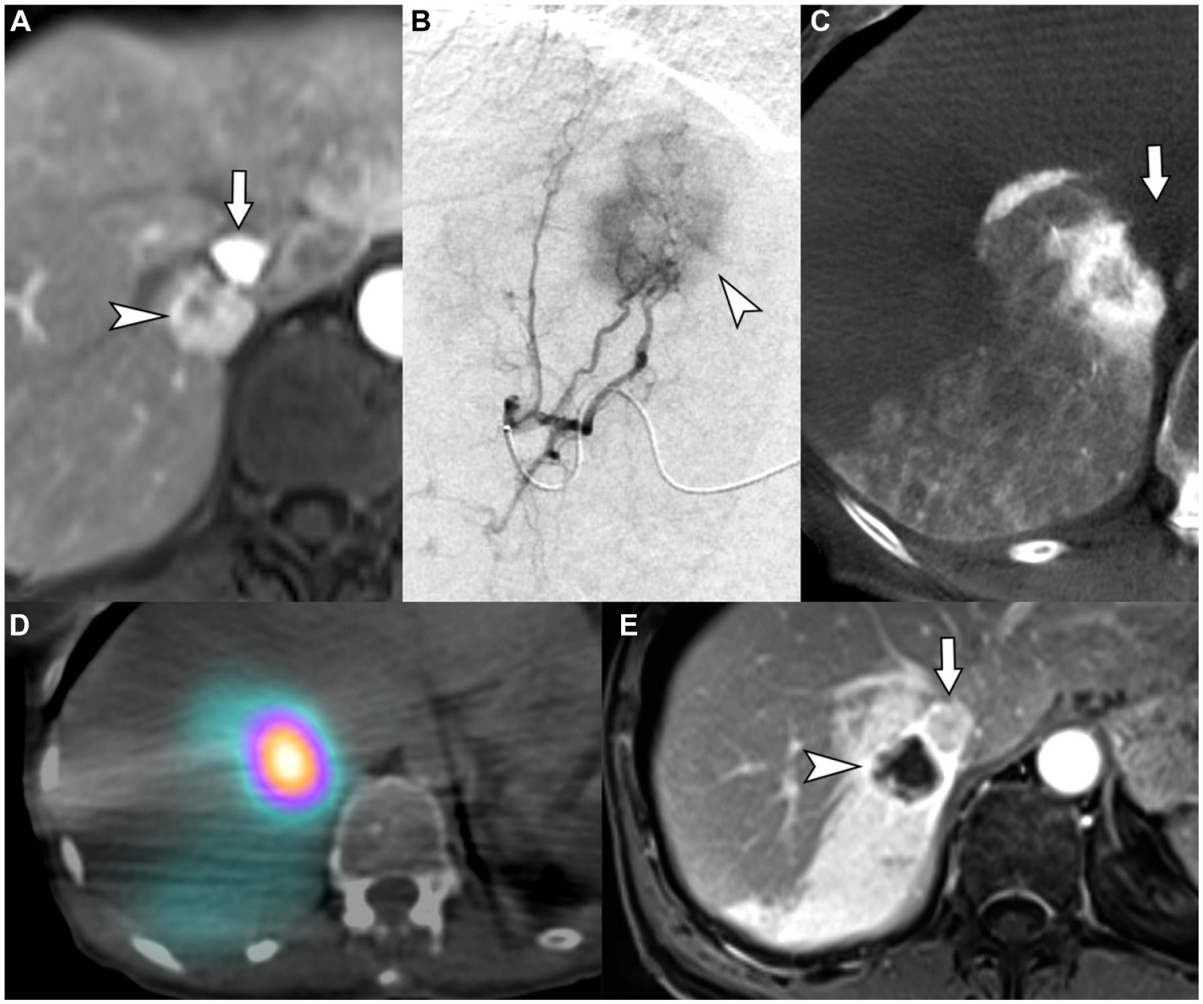
62-year-old female with intrahepatic cholangiocarcinoma in need for liver therapy as a bridge to hepatic resection while being treated for an unrelated second primary adenocarcinoma of lung. (**A**) Contrast-enhanced MRI demonstrates an iCCA (arrowhead) that abuts the right hepatic vein and intrahepatic inferior vena cava (arrow). (**B**) Segment VII selective arteriogram demonstrating tumor hyper-enhancement (arrowhead). (**C**) Cone-beam computed tomography demonstrating complete coverage of the hyper-enhancing tumor and margins within the segment VII angiosome (arrow indicates the non-enhancing inferior vena cava). (**D**) Bremsstrahlung SPECT/CT after radioembolization of the segment VII artery with a dose of 256.6 Gy demonstrates well-circumscribed high tumor-to-parenchyma uptake surrounded by lower uptake of radioactivity in segment VII expendable volumes of liver, illustrating favorable macrovascular and microvascular conduit. (**E**) Contrast-enhanced magnetic resonance imaging 6 months after radioembolization showing complete response in the tumor, with decrease in size and lack of internal enhancement (arrowhead) (arrow indicates the IVC). The patient underwent liver resection one year later with histologic analysis demonstrating complete pathologic necrosis of tumor.

**Figure 7:**
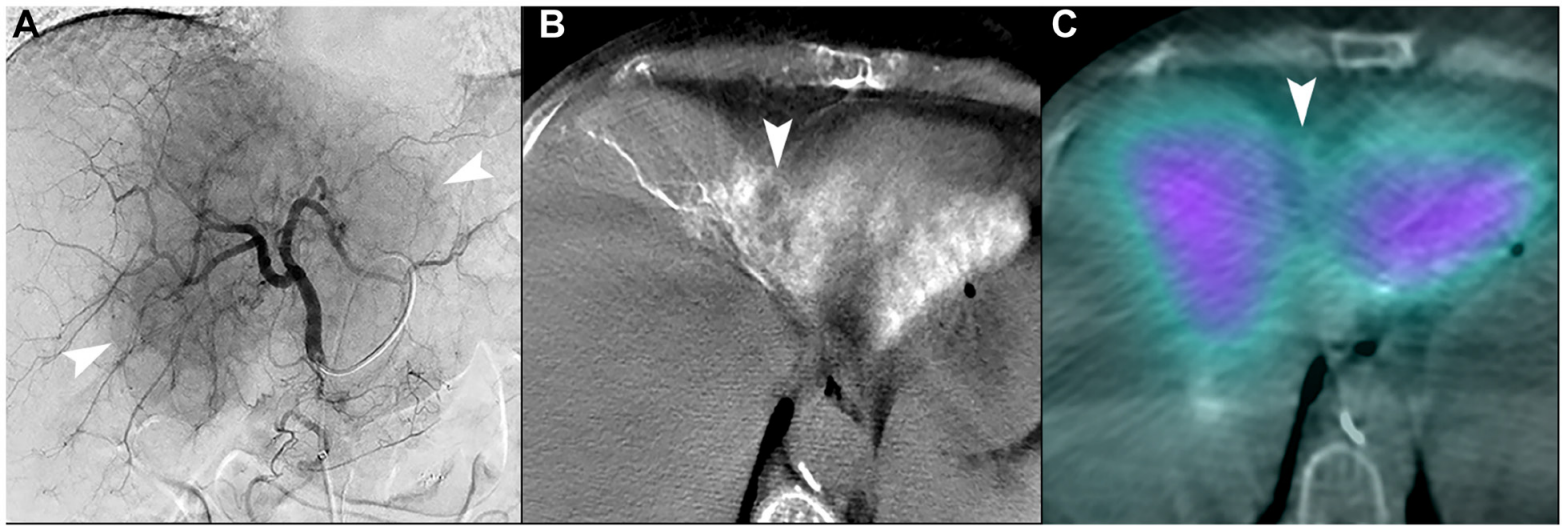
Unfavorable macrovascular and microvascular arterial conduit in two patients with intrahepatic cholangiocarcinoma. (**A**) Single image from proper hepatic arteriogram shows a large hypervascular iCCA (arrowheads). Cone-beam computed tomography of multiple branches (not shown) demonstrated that tumor blood supply would require treatment of non-expendable liver, which could not be adequately attenuated with distal angiosomal truncation representing unfavorable macrovascular conduit. Ablative intent radioembolization was not offered. (**B**) Cone-beam computed tomography of a different patient shows an iCCA in segment IVA with poor enhancement of tumor (arrowhead). (**C**) Bremsstrahlung SPECT/CT after radioembolization showed poor uptake of microspheres in the tumor (arrowhead), representing an unfavorable microvascular conduit.

Table 1 shows the distribution of vascular conduit quality. The unfavorable conduit group had a significantly worse OS, HR 5.7 (95% CI 1.1–28.9, *p* = 0.034), and PFS, HR 5.9 (95% CI 1.9–18.4, *p* = 0.002), compared to the group with favorable macro- and microvascular conduit (Table 5 and Figures 3 and 4). Figures 5, 6 and 7 present clinical examples of favorable and unfavorable vascular conduit.

## DISCUSSION

Radioembolization techniques have evolved over the past decade to increase tumor dose while reducing the volume of radiation to uninvolved liver. Following basic principles of radiation biology, this approach has led to improved outcomes when used for the treatment of hepatocellular carcinoma where MIRD doses >190 Gy have resulted in increased tumor pathologic necrosis [7]. Similarly, external beam radiation has shown improved outcomes when ablative doses can be achieved for cholangiocarcinoma [9]. However, most iCCA radioembolization studies have not explored the use of radioembolization with ablative intent.

This study evaluated patients with iCCA treated with angiosome-based, glass microsphere radioembolization using at least one single compartment MIRD dose of >190 Gy to greater than 75% of the tumor burden. Adverse events were low (7.1% grade 3 and 4), and there was no significant change in hepatic function or performance status 3 months after radioembolization, or procedure related mortality. Initial efficacy demonstrated an objective response rate of 94.1% (CR of 44.1%) and freedom from in-field lesion progression of 79% at 24 months. Median overall survival was not reached and was 59% at 30 months.

Our institution has previously published outcomes on the treatment of unresectable iCCA treated with resin microspheres using body surface area (BSA) dosimetry. In contrast to the currently presented outcomes using ablative radioembolization, the prior study demonstrated a modified Response Evaluation Criteria in Solid Tumors (mRECIST) response rate of 36.4% (all PR), a median survival of 9 months, and a 30-month survival of 20.4% [10]. Although the current study could not be designed as a direct comparison to our former experience, the discrepancy in outcomes for similar populations suggests favoring the treatment of iCCA with ablative intent radioembolization instead of BSA dosimetry.

Reported outcomes for radioembolization treated with conventional body surface area or ≤ 150 Gy single compartment radioembolization dose methodologies have been varied. A multi-institutional study found a 3-year OS rate of 4% using resin and glass microspheres, with most receiving resin microspheres with BSA dosimetry; single compartment MIRD doses were not reported [11]. In a retrospective study, patients treated with glass microspheres and a mean tumor dose of ≥ 150 Gy demonstrated improved OS over patients who received < 150 Gy (*p* = 0.031) [12]. In a larger retrospective single center study of patients with biliary tract cancer receiving glass microsphere radioembolization, median OS was significantly higher in tumors treated with ≥ 260 Gy compared to < 260 Gy (28.2 vs 11.4 months, *p* = 0.019), supporting that outcomes of radioembolization for iCCA are dose dependent [13]. A recent phase 2 clinical trial of first-line glass microsphere radioembolization with concurrent gemcitabine and cisplatin utilized dose personalization to achieve a median tumor of 317 Gy and demonstrated a median overall survival of 22 months and 45% at 24 months [14].

As cholangiocarcinoma is known to present with varied phenotypes, our study evaluated the quality of arterial supply to tumors in the form of a conduit analysis. Given that tumors were initially unresectable, the majority involved multiple segments and often in the central portion of the liver. Conceptually, the quality of micro- and macrovascular conduit is related to the ability of trans-arterial brachytherapy to deposit within tumor. Favorable microvascular and macrovascular conduit in this study were associated with the best outcomes. In a retrospective study of patients with iCCA treated with resection, the 5-year risk of death in patients with diffuse tumor hyperenhancement per preoperative magnetic resonance imaging was lower when compared to tumors with either peripheral enhancement or diffuse hypoenhancement (5-year risk of death: 5.9% vs 59.2% vs 87.9%) [8]. Our study similarly identified hypoenhancement (unfavorable microvascular conduit) as a negative marker for survival with HR 5.7 (95% CI 1.1–28.9, *p* = 0.034).

Gemcitabine and cisplatin combination is the current systemic therapy standard of care for patients with iCCA. The landmark Phase 3 trial for these agents yielded a median OS of 11.7 months in favor of gemcitabine and cisplatin versus gemcitabine alone [15]. Advancements in targeted therapy have shown promising result in tumors with actionable mutations, however, these are present only in a minority of patients and have Grade 3 or greater adverse events, which have been reported in excess of 40% in phase 2 studies [16, 17]. While this study was not designed to analyze the efficacy of systemic therapy in conjunction with radioembolization, the authors believe that both therapies can be safely provided in select patients with potential synergy.

Neoadjuvant radioembolization has been reported for the treatment of initially unresectable iCCA with successful conversion to resection candidacy in 22% of patients [14]. In a phase 3 trial of surgical resection of heterogeneous biliary tract cancers with and without adjuvant capecitabine, the per-protocol analysis yielded a median overall survival of 53 months and 36 months, respectively (*p* = 0·028) [18]. In another phase 3 randomized trial comparing surgical resection of biliary tract cancers with and without adjuvant gemcitabine and oxaliplatin, overall survival was not different, although adverse events were significantly greater for the systemic therapy arm (grade 3 in 62% versus 18% and grade 4 in 11% versus 3% (*P* < 0.001) [19]. In a retrospective study or patients receiving neoadjuvant lobar radioembolization, which included patients with intrahepatic cholangiocarcinoma, the rate of post hepatectomy liver failure was 3.8% [20]. Ultimately, radioembolization has demonstrated the capability of converting initially unresectable patients to curative intent resection, and both therapies can be used as part of an aggressive care plan for select patients with locally advanced iCCA.

The limitations of the present study include the small sample size and retrospective nature. This study only included patients with iCCA and excluded other biliary tract cancers with potentially worse prognoses. Only patients who were candidates for ablative radioembolization were included. Patients who received doses greater than 190 Gy were included, but no comparison was made with patients with similar disease that received lower doses. An intention-to-treat analysis was not performed, as this was outside the scope of the exploratory design of this study. This study did not adequately evaluate whether radioembolization can be employed as a sole therapy in patients with unresectable tumors. Response rates were measured using mRECIST which has not been extensively studied for iCCA. An analysis of cross-sectional imaging features and the impact on response was not performed. Compartment and voxel dosimetry analyses were not performed because complete data was not available. Lastly, the concept of vascular conduit quality, as applied clinically by the interventional radiologists that participated in this study, has not been externally validated.

## MATERIALS AND METHODS

### Clinical setting and study population

Institutional Review Board approval was obtained for this minimal risk, retrospective review of consecutive patients conducted between 5/12/2016 and 2/14/2020. The need for informed consent was waived. Data collected included demographics, Eastern Cooperative Oncology Group (ECOG) performance status, etiology of liver disease, laboratory values, tumor characteristics obtained from multiphase contrasted MRI or CT imaging, and biopsy results. Tumors were categorized according to three morphologic subtypes, mass-forming, periductal-infiltrating and intraductal [21, 22]. The Albumin-Bilirubin (ALBI) score, Model for End-Stage Liver Disease score (MELD), and Child-Pugh (CP) class were calculated. Tumors were staged using the 8th edition of the American Joint Committee on Cancer (AJCC) Staging Manual [23]. Treatment consensus was met after formal presentation at an interdisciplinary hepatobiliary tumor board with one or more liver surgeons who determined tumor resectability. The dosimetry details of all patients with biopsy and imaging confirmation of iCCA treated with radioembolization were reviewed. Those who did not receive a single or cumulative absorbed dose >190 Gy in at least one tumor-containing angiosome and to > 75% of tumor volume were excluded.

### Prior, concurrent, and post-radioembolization systemic therapy

Prior systemic therapy was defined as chemotherapy, targeted agent, or immunotherapy in which the treatment regimen was stopped at least 45 days before radioembolization. Concurrent therapy was defined as treatment received within 45 days, before or after, radioembolization. Post-procedure therapy was defined as treatment received 45 days after radioembolization. Previous and post-treatment locoregional and surgical therapies were tabulated.

### Arterial mapping and radioembolization

Pre-procedural hepatic angiography with cone-beam CT was performed in all patients to map the angiosomes that provided tumor blood supply. Intra-arterial technetium-99m macroaggregated albumin (^99m^Tc-MAA) administration was performed in each patient, followed by SPECT/CT. Treatment considerations were made based on performance status, hepatic function, tumor focality and volume, and potential volume of liver exposed to radiation. Single compartment MIRD methodology, which assumes uniform distribution of activity within the treatment volume, was used to calculate dosimetry [24]. Microsphere administration was defined as either segmental (less than one lobe) or lobar. Dosimetry was determined by the authorized user interventional radiologist based on mapping angiography findings, cone-beam computed tomography (CBCT) anatomic volumes, ^99m^Tc-MAA SPECT/CT, and laboratory values. Treatment volumes were calculated with Visage version 7.1 (Visage Imaging Inc, San Diego, CA) using the freehand 3-dimensional region of interest function along the contrast-enhancing angiosomal boundaries, as shown in the CBCT axial images, and expressed in cm^3^. In general, liver deemed expendable by the treating physician was dosed >190 Gy with a reduction applied to patients with limited liver reserve. ^90^Y-containing glass microspheres (TheraSphere; Boston Scientific, Marlborough, MA, USA) were used for all radioembolization treatments, followed by bremsstrahlung SPECT/CT. All procedures were performed on an outpatient basis with discharge after a 2-hour observation.

### Tumor arterial supply qualitative analysis

The arterial supply to tumors (arterial conduit) was qualitatively analyzed for the presence of favorable microvascular conduit (high intrinsic tumor vascularity as demonstrated with contrast-enhancement during angiography, CBCT, and ^99m^Tc-MAA uptake), and favorable macrovascular conduit (arterial supply amenable to selective catheterization that included expendable hepatic parenchyma) [25]. The data was analyzed in two groups, one included those with favorable macro- and microvascular conduit, and the other combined those with any type of unfavorable macro- or microvascular conduit.

### Clinical and imaging follow-up

Follow up at 1-month after radioembolization, and every 3 months thereafter, included serologic analysis, contrast-enhanced multiphase magnetic resonance imaging or computed tomography. Assessment of ECOG performance status, ALBI score, MELD score, and CP score was done at 3 and 6 months after radioembolization. Target-lesion response by mRECIST at 3 months was analyzed under supervision of board-certified radiologists with 8–30 years of experience [26]. Tumor progression was categorized as either within the radiation field (in-field), or outside of the radiation field (out-of-field). Adverse events were reported using the Common Terminology Criteria for Adverse Events (CTCAE) version 5.0 [27].

### Statistical analysis

A Wilcoxon signed-rank test was used to evaluate the change in posttreatment CA 19-9 levels at 3 months in those who presented with an elevated value (≥ 35 U/mL). The McNemar test was used to evaluate the change in ALBI, MELD, and CP categories, as well as the ECOG status before and after treatment at 3 months. The Kaplan-Meier method was used to estimate OS, PFS, and FFP. Subsequent surgery was considered a censoring event. Univariate Cox regression analysis was conducted to determine effect of tumor morphology and quality of vascular conduit on the overall and progression-free survival. A *p*-value ≤ 0.05 was considered statistically significant. All analyses were conducted using SPSS software (SPSS, Version 25.0. Armonk, NY: IBM Corp.).

## CONCLUSIONS

Angiosome-based radioembolization using a single compartment MIRD dose of >190 Gy for the treatment of unresectable intrahepatic cholangiocarcinoma has a low incidence of adverse events, high rates of response, and an overall survival of 59% at 30 months.

## Abbreviations

iCCA: Intrahepatic cholangiocarcinoma
ALBI: Albumin-Bilirubin
MELD: Model for End-Stage Liver Disease
CP: Child-Pugh
ECOG: Eastern Cooperative Oncology Group
SPECT: Single photon emission computed tomography
^99m^Tc-MAA: Technetium-99m macroaggregated albumin
^90^Y: Yttrium-90
Gy: Gray
GBq: Giga Becquerel
AJCC: American Joint Committee on Cancer
MIRD: Medical Internal Radiation Dose
mRECIST: modified Response Evaluation Criteria In Solid Tumors
CTCAE: Common Terminology Criteria for Adverse Events
OS: Overall survival
PFS: Progression free survival
FFP: Freedom from Progression
KM: Kaplan-Meier
CR: Complete Response
PR: Partial Response
SD: Stable Disease
PD: Progression of Disease
HR: Hazard Ratio
CI: Confidence Interval
CBCT: cone-beam computed tomography
